# Minor envelope proteins from GP2a to GP4 contribute to the spread pattern and yield of type 2 PRRSV in MARC-145 cells

**DOI:** 10.3389/fcimb.2024.1376725

**Published:** 2024-03-25

**Authors:** Yuan-Zhe Bai, Yue Sun, Yong-Gang Liu, Hong-Liang Zhang, Tong-Qing An, Qian Wang, Zhi-Jun Tian, Xinyuan Qiao, Xue-Hui Cai, Yan-Dong Tang

**Affiliations:** ^1^ State Key Laboratory for Animal Disease Control and Prevention, Harbin Veterinary Research Institute of Chinese Academy of Agricultural Sciences, Harbin, China; ^2^ Heilongjiang Key Laboratory for Animal Disease Control and Pharmaceutical Development, Department of Preventive Veterinary Medicine, College of Veterinary Medicine, Northeast Agricultural University, Harbin, China; ^3^ Harbin Veterinary Research Institute, Heilongjiang Provincial Research Center for Veterinary Biomedicine, Harbin, China; ^4^ Harbin Veterinary Research Institute, Heilongjiang Provincial Key Laboratory of Veterinary Immunology, Harbin, China

**Keywords:** spread pattern, PRRSV, cell-to-cell, cell-free, yield

## Abstract

In China, porcine reproductive and respiratory syndrome virus (PRRSV) vaccines are widely used. These vaccines, which contain inactivated and live attenuated vaccines (LAVs), are produced by MARC-145 cells derived from the monkey kidney cell line. However, some PRRSV strains in MARC-145 cells have a low yield. Here, we used two type 2 PRRSV strains (CH-1R and HuN4) to identify the genes responsible for virus yield in MARC-145 cells. Our findings indicate that the two viruses have different spread patterns, which ultimately determine their yield. By replacing the viral envelope genes with a reverse genetics system, we discovered that the minor envelope proteins, from GP2a to GP4, play a crucial role in determining the spread pattern and yield of type 2 PRRSV in MARC-145 cells. The cell-free transmission pattern of type 2 PRRSV appears to be more efficient than the cell-to-cell transmission pattern. Overall, these findings suggest that GP2a to GP4 contributes to the spread pattern and yield of type 2 PRRSV.

## Introduction

Porcine reproductive and respiratory syndrome viruses (PRRSVs) are important swine pathogens, and outbreaks of these viruses have significant economic impacts on the swine industry (Meulenberg, 2000). PRRSVs are typically categorized into two species: PRRSV-1, which is the type 1 genotype of European origin, and PRRSV-2, which is the type 2 genotype of North American origin. PRRSV-1 and PRRSV-2 are classified as arteriviruses and are in the order *Nidovirales* (Gorbalenya et al., 2006). PRRSV exhibits highly restricted cell tropism, specifically targeting differentiated and activated monocyte/macrophage lineages (Duan et al., 1997). PRRSV-1 and PRRSV-2 were successfully isolated from porcine alveolar macrophages (PAMs) (Wensvoort et al., 1991; Yim-Im et al., 2021). However, there are limitations to virus isolation and propagation in PAMs. These limitations include the short lifespan of primary cells, which require periodic preparation; variations in quality; heterogeneity in cell populations; and a high risk of contamination. Additionally, the procedures for PAM preparation are laborious and technically challenging (Yim-Im et al., 2021). MARC-145 cells, a subclone originating from the monkey kidney cell line MA-104, have been shown to be permissive to PRRSV replication (Kim et al., 1993). The MARC-145 cell line has become the most commonly used cell line for PRRSV studies in most laboratories. In China, most commercial PRRSV vaccines are propagated in MARC-145 cells. Therefore, the yield of PRRSV in MARC-145 cells is very important for vaccine development, particularly for inactivated vaccines.

Enveloped viruses primarily infect target cells through two pathways. The first pathway involves cell-free viruses attaching to cellular receptors and being taken into cells through endocytosis. Once inside the cell, the viral envelope fuses with the endosomal membrane, releasing the viral capsid into the cytosol. The second pathway involves the virus entering adjacent cells via a cell-to-cell model, which mainly involves inducing membrane fusion between infected and uninfected cells, allowing the viral genetic material to enter the uninfected cell and complete replication (Zhong et al., 2013; Yang et al., 2020; Yang et al., 2021). PRRSV was found to transport viral RNA and proteins into adjacent cells via a cell-to-cell pathway. This mode of viral transmission relies on the interaction between certain viral proteins and cytoskeletal proteins (Cafruny et al., 2006; Guo et al., 2016). However, which gene(s) determine the spread pattern of cell-to-cell is unclear. In the present study, we utilized two different strains of type 2 PRRSV to examine how the virus spreads and replicates in MARC-145 cells. Using a viral reverse genetics platform, we found that the minor envelope proteins, specifically GP2a to GP4, are essential for determining the spread pattern and yield of type 2 PRRSV in MARC-145 cells. This work will be helpful for PRRSV vaccine development.

## Materials and methods

### Cells, viruses, and antibodies

MARC-145 cells were stored in our lab. The highly pathogenic PRRSV strain HuN4 was rescued from the PRRSV HuN4 infectious clone (PRRSV HuN4-F5) (Tang et al., 2016; Wang et al., 2019). The PRRSV vaccine strain CH-1R was rescued from the PRRSV CH-1R infectious clone constructed via the same strategy as the PRRSV HuN4-F5 infectious clone. PRRSV N antibodies were prepared in our laboratory.

### Chimeric PRRSV construction

CH-1R and HuN4 infectious clones were used as backbones to construct chimeric PRRSVs as our previous work (Zhang et al., 2018).

### Growth kinetics

MARC-145 cells were infected with the indicated viruses at an multiplicity of infection (MOI) of 0.01 at 4°C. After 2 hours, the cells were washed three times with cold PBS to remove the unbound viruses. Fresh medium supplemented with 2% FBS was added to the cells. The medium supernatants were harvested and stored at -80°C at the indicated times post infection. The virus samples were then titrated on MARC-145 cells using the 50% tissue culture infective dose (TCID_50_) method.

### Immunofluorescence assay

The IFA was performed as described in our previous reports (Zhang et al., 2021; Chen et al., 2022; Zhang et al., 2022). Briefly, MARC-145 cells were infected with the indicated viruses and then fixed with 4% paraformaldehyde (PFA). Then, the cells were permeabilized with 0.5% Triton X-100. After that, the cells were blocked with 2% bovine serum albumin (BSA) and then labeled with antibodies. The cell nucleus was finally stained with 4’,6-diamino-2-phenylindole (DAPI). The fluorescence signals were then detected using a fluorescence microscope.

### Viral load in cells and supernatant

MARC-145 cells were seeded on 6-cell plates and infected with the indicated viruses at an MOI of 0.01. At 36 h post infection, the supernatant was harvested, and the cells were scraped down with a cell scraper and diluted to the same volume of medium as the supernatant. Viral samples from the supernatants and intracellular fractions were frozen and thawed once and subsequently stored at -80°C. The virus samples were titrated on MARC-145 cells by TCID_50_.

### Transwell coculture system

Cell-to-cell transmission assays were performed as described in our recent work (Yang et al., 2020). MARC-145 cells were plated onto permeable filters (Corning, 6.5 mm, pore size 0.4 µm) at a density of 1 × 10^5^ cells. When the cells grew to a tightly connected state, they were infected with the indicated viruses at an MOI of 0.01 for 2 h at 4°C. These virus-infected cells were used as effector cells. The filters were suspended in wells in a 24-well plate already containing target cells. After 36 h, the effector cells and target cells were fixed, permeabilized, and subjected to IFA.

### Statistical analysis

Statistical analysis was conducted using GraphPad Prism 8.0 software. The statistical significance of the differences was analyzed using t tests. A P value < 0.05 was considered to indicate statistical significance.

## Results

### The HuN4 and CH-1R strains exhibited distinct spreading patterns and yields on MARC-145 cells

**Figure 1:**
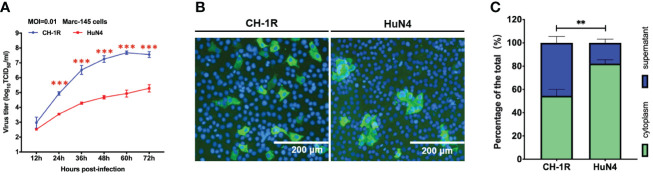
CH-1R and HuN4 exhibit distinct yields and spread patterns on MARC-145 cells. **(A)** MARC-145 cells were infected with CH-1R or HuN4 at an MOI of 0.01. At the indicated time post infection, the supernatant was harvested and titrated. An asterisk (*) indicates a significant difference between HuN4 and CH-1R (**p < 0.01; ***p < 0.001). **(B)** Infected cells at 36 hours post infection were detected via an immunofluorescence assay. Scale bars, 200 μm. **(C)** MARC-145 cells were infected with CH-1R or HuN4 at an MOI of 0.01. At 36 hours postinfection, the cells and supernatant were harvested and titrated on MARC-145 cells. The percentage of the total viral load in the supernatant and cells was calculated.

**Figure 2:**
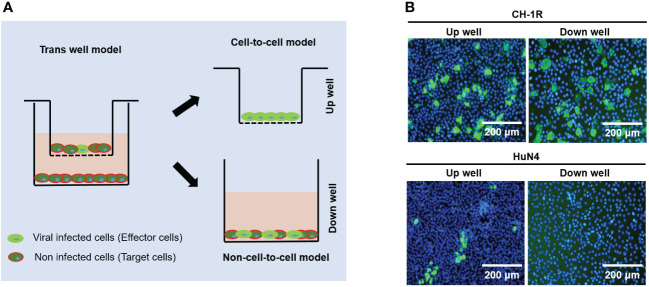
The spreading pattern of PRRSV was verified by a transwell coculture system. **(A)** Experimental design of a transwell coculture system. MARC-145 cells were seeded on transwell filters and preinfected with PRRSV as effector cells. Uninfected MARC-145 cells were seeded in 24-well plates as target cells. **(B)** Effector cells were infected with CH-1R or HuN4 at an MOI of 0.01. At 36 hours postinfection, the cells were fixed, permeabilized, and subjected to an immunofluorescence assay. Scale bars, 200 μm.

CH-1R, a commercial live attenuated vaccine in China, was developed by successive passaging on MARC-145 cells with CH-1a for approximately 160 generations. HuN4 is a highly pathogenic PRRSV strain isolated in China (Tong et al., 2007). MARC-145 cells were infected with CH-1R or HuN4 (MOI=0.01). The cell culture supernatants were collected and titrated at the indicated time points post infection. The replicative growth curves of CH-1R and HuN4 MARC-145 cells were analyzed (**Figure 1A**). The yield of CH-1R on MARC-145 was significantly greater than that on HuN4. Interestingly, by IFA, we found that the HuN4 and CH-1R strains exhibited distinct spreading patterns (**Figure 1B**). MARC-145 cells infected with the HuN4 strain are characterized by the formation of infected cell clusters. This finding suggested that cell-to-cell spread originated from a single PRRSV-infected cell, which has also been reported by other groups (Cafruny et al., 2006; Guo et al., 2016). However, for the CH-1R strain, the infected cells were distributed in a scattered manner. Therefore, we hypothesized that CH-1R may have a distinct spread pattern. To verify this hypothesis, MARC-145 cells were infected with CH-1R or HuN4 (MOI=0.01), and 36 h post infection, intracellular and supernatant infectious viral particles were examined. The percentage of viral titer in the cell and the supernatant was calculated. We found that the percentage of viral particles in the supernatants of cells infected with CH-1R was significantly greater than that in the supernatants of cells infected with HuN4 (**Figure 1C**). This result suggested that, compared to HuN4, CH-1R is more favorable as a cell-free infection mode rather than a cell-to-cell infection mode. To further confirm this, we used a transwell coculture system, as illustrated in **Figure 2A**. The effector cells were preinfected with HuN4 or CH-1R at an MOI of 0.01 and cocultured with the target cells. At 36 h post infection, the effector cells and target cells were fixed and subjected to IFA. We found that a large number of MARC-145 cells in the CH-1R infection group were infected with the target cells. However, for HuN4, only sporadic virus-infected cells were able to be detected among the target cells (**Figure 2B**). Overall, the HuN4 and CH-1R strains exhibited distinct spreading patterns on MARC-145 cells.

### Envelope proteins contribute to the yield and spread pattern of MARC-145 cells

**Figure 3:**
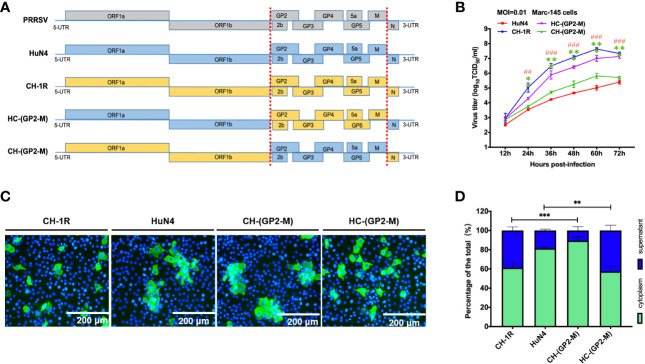
Envelope proteins determine the PRRSV spread pattern and yield. **(A)** Schematic diagram of chimeric PRRSV recombinants. **(B)** MARC-145 cells were infected with indicated virus at an MOI of 0.01. At 36 hours postinfection, the infected cells were detected by IFA. Scale bars, 200 μm. **(C)** MARC-145 cells were infected with the indicated viruses at an MOI of 0.01. At the indicated time points postinfection, the cell culture supernatant was harvested and titrated on MARC-145 cells. Asterisk (*) indicates a significant difference between CH-1R and CH-(GP2-M) (**p < 0.01; ***p < 0.001). Pound (^#^) indicates a significant difference between HuN4 and HC-(GP2-M) (^##^p < 0.01; ^###^p < 0.001). **(D)** MARC-145 cells were infected with the indicated viruses at an MOI of 0.01. At 36 hours post infection, the cells and culture supernatant were harvested, and titrated on MARC-145 cells. The percentages of the total viral load in the supernatant and in the cytoplasm were calculated.

Viral yield and spread patterns are always determined by viral envelope proteins. To determine which viral genes are responsible for these patterns, we started by constructing chimeric PRRSV recombinants and substituting the corresponding viral envelope genes with those from CH-1R and HuN4 (**Figure 3A**). After CH-(GP2-M) and HC-(GP2-M) were rescued, the MARC-145 cells were infected with CH-1R, HuN4, CH-(GP2-M), or HC-(GP2-M) at an MOI of 0.01. At 36 hours postinfection, the cells were fixed and subjected to IFA. MARC-145 cells infected with HC-(GP2-M) exhibited a spread pattern similar to that of CH-1R. In contrast, the spread pattern of HC-(GP2-M) in MARC-145 cells was comparable to that in cells infected with HuN4 (**Figure 3B**). Next, we evaluated the yields of CH-(GP2-M) and HC-(GP2-M) in MARC-145 cells. CH-1R and HuN4 were used as controls. We found that at 24 hours postinfection, the viral titer of the HC-(GP2-M) was significantly greater than that of the HuN4 individuals. In contrast, the viral growth of CH-(GP2-M) was markedly reduced compared to that of CH-1R (**Figure 3C**). The percentage of the viral load in the supernatant and cytoplasm was also evaluated (**Figure 3D**). These results indicated that the envelope proteins from GP2 to M determines the viral yield and spread pattern of CH-1R and HuN4 on MARC-145 cells.

### The minor envelope proteins GP2a to GP4 determine the viral yield and spread pattern in MARC-145 cells

**Figure 4:**
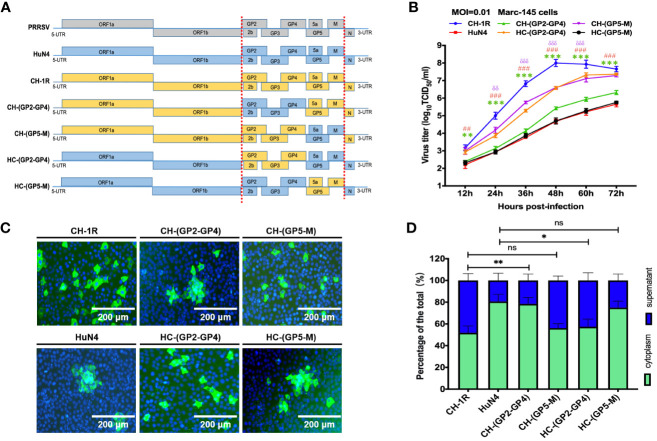
The presence of minor viral envelope proteins ranging from GP2a to GP4 contributes to the yield and spread pattern of PRRSV. **(A)** Schematic diagram of chimeric PRRSV recombinants. **(B)** MARC-145 cells were infected with indicated virus at an MOI of 0.01. At 36 hours postinfection, the cells were subjected to IFA. Scale bars, 200 μm. **(C)** MARC-145 cells were infected with the indicated viruses at an MOI of 0.01. At the indicated time points postinfection, the cell culture supernatant was harvested and titrated on MARC-145 cells. Asterisk (*) indicates a significant difference between CH-1R and CH-(GP2-GP4) (**p < 0.01; ***p < 0.001). Pound (^#^) indicates a significant difference between HuN4 and HC-(GP2-GP4) (^##^p < 0.01; ^###^p < 0.001). Delta (δ) indicates a significant difference between CH-1R and CH-(GP5-M) (δδ: p < 0.01; δδδ: p < 0.001). **(D)** MARC-145 cells were infected with the indicated viruses at an MOI of 0.01. At 36 hours postinfection, the cells and cell culture supernatant were harvested, and titrated on MARC-145 cells. The percentages of the total viral load in the supernatant and in the cytoplasm were calculated. ns, no significance.

We next further investigated which viral proteins impact the yield and spread pattern of PRRSV. CH-1R and HuN4 were employed as the parental strains, with their genes being swapped for major envelope proteins and minor envelope proteins, respectively (**Figure 4A**). The chimeric viruses CH-(GP2-GP4), CH-(GP5-M), HC-(GP2-GP4), and HC-(GP5-M) were successfully rescued in MARC-145 cells. MARC-145 cells were infected with these chimeric mutant viruses at an MOI of 0.01. At 36 h postinfection, the cells were fixed and subjected to IFA. We found that MARC-145 cells infected with CH-(GP5-M) or HC-(GP2-GP4) were scattered, which is in line with what has been observed for CH-1R. Furthermore, MARC-145 cells were infected with CH-(GP5-M) or HC-(GP2-GP4) were clustered, which was in line with what was observed for HuN4 (**Figure 4B**). Furthermore, compared with those of CH-1R, the viral yields of CH-(GP5-M) were slightly impaired; however, the viral yields of CH-(GP2-GP4) were significantly impaired compared to those of CH-1R. Additionally, the HC-(GP2-GP4) produced more progeny from MARC-145 cells than from HuN4 cells (**Figure 4C**). Finally, we quantified the viral infectious particles in the supernatant and cytoplasm (**Figure 4D**). The results showed that minor envelope proteins from GP2a to GP4 were able to determine the pattern of PRRSV spread in MARC-145 cells.

## Discussion

In the MARC-145 cell line, PRRSV infection has been shown to spread via cell-to-cell transmission (Cafruny et al., 2006). Furthermore, subsequent studies have shown that PRRSV utilizes the cytoskeletal machinery of host cells for efficient cell-to-cell spread (Guo et al., 2016). Research has shown that PRRSV is transmitted between cells via intercellular nanotubes that contain filamentous actin (F-actin) and myosin-associated motor proteins. The use of drugs that block actin polymerization or myosin IIA activation prevents the formation of nanotubes and viral clusters in infected cells (Guo et al., 2016). Furthermore, F-actin and myosin IIA coprecipitated with PRRSV nsp1β, nsp2, nsp2TF, nsp4, nsp7-nsp8, GP5, and N proteins (Guo et al., 2016). However, our research indicated that the minor envelopment protein GP2a to GP4 contributes to the spread pattern of PRRSV. Therefore, further investigations are needed to determine whether these viral envelope proteins interact with or utilize the host cell cytoskeletal machinery for efficient cell-to-cell spread. In fact, developing a safe and effective PRRSV vaccine is an enormous challenge, especially when faced with a heterogeneous virus (Thanawongnuwech et al., 2010; Nan et al., 2017). Novel technologies used for other viruses may be applied for the development of a PRRSV vaccine in the future (Wang et al., 2022; Tang et al., 2023; Wang et al., 2023). However, regardless of the technology used, the yield of the vaccine was very important. In this study, we discovered that GP2a to GP4 contributes to the spread pattern of type 2 PRRSV. We did not replace a single gene in this region because the genes in this region overlapped. Therefore, we cannot delineate the phenotype or viral-specific gene function. In our previous work, we also found that field PRRSVs in MARC-145 cells were determined by variations in the minor envelope protein GP2a-GP3 (Zhang et al., 2018). In fact, identifying which specific gene contributes to the cell-to-cell spread pattern is difficult (unpublished data). GP2, GP3, and GP4 may form a complex and may work together to determine the degree of viral spread in MARC-145 cells. Due to the interaction of GP2 and GP4 with the PRRSV receptor CD163, we speculate that the cell-to-cell spread pattern may also be correlated with their interaction with CD163 (Welch and Calvert, 2010; Zhang and Yoo, 2015). The abundance of CD163 may also be a determining factor for the spread pattern of this disease (Wang et al., 2018). Taken together, the results of this study revealed that GP2a to GP4 contributes to the spread pattern of type 2 PRRSV, determining its cell-to-cell or cell-free transmission, and is correlated with virus yield. GP5 and M also have an impact on viral yield, but not on the spread pattern. In our study, the cell-free transmission pattern of type 2 PRRSV appeared to be more efficient than the cell-to-cell transmission pattern. However, it is important to further explore whether other type 2 PRRSV strains also exhibit this phenomenon. Overall, our findings provide new insight into the lifecycle of PRRSV and a new way to increase the titer of PRRSV in MARC-145 cells.

## Data availability statement

The original contributions presented in the study are included in the article/supplementary material. Further inquiries can be directed to the corresponding authors.

## Author contributions

Y-ZB: Data curation, Formal analysis, Investigation, Validation, Visualization, Writing – original draft. YS: Data curation, Formal analysis, Methodology, Resources, Software, Validation, Writing – original draft. Y-GL: Data curation, Formal analysis, Investigation, Methodology, Project administration, Resources, Software, Supervision, Writing – original draft. H-LZ: Data curation, Formal analysis, Investigation, Methodology, Resources, Software, Writing – original draft. T-QA: Conceptualization, Supervision, Writing – review & editing. QW: Resources, Writing – review & editing. Z-JT: Resources, Writing – review & editing. XQ: Conceptualization, Supervision, Writing – review & editing. X-HC: Conceptualization, Supervision, Writing – review & editing. Y-DT: Conceptualization, Funding acquisition, Writing – review & editing.
